# Cardiac Tissue Bioprinting: Integrating Structure and Functions Through Biomimetic Design, Bioinks, and Stimulation

**DOI:** 10.3390/gels11080593

**Published:** 2025-07-31

**Authors:** Silvia Marino, Reem Alheijailan, Rita Alonaizan, Stefano Gabetti, Diana Massai, Maurizio Pesce

**Affiliations:** 1 Discipline of Mechanical, Manufacturing and Biomedical Engineering, Trinity College Dublin, D02 PN40 Dublin, Ireland; marinos@tcd.ie; 2 Trinity Centre for Biomedical Engineering, Trinity Biomedical Science Institute, Trinity College Dublin, D02 R590 Dublin, Ireland; 3 King Faisal Specialist Hospital & Research Center, Riyadh 12713, Saudi Arabia; rhijailan@kfshrc.edu.sa (R.A.); ralonaizan@kfshrc.edu.sa (R.A.); 4 PolitoBIOMed Lab and Department of Mechanical and Aerospace Engineering, Politecnico di Torino, 10129 Turin, Italy; stefano.gabetti@polito.it (S.G.); diana.massai@polito.it (D.M.); 5 Interuniversity Center for the Promotion of the 3Rs Principles in Teaching and Research, 10129 Turin, Italy; 6 College of Medicine, Alfaisal University, Riyadh 11533, Saudi Arabia

**Keywords:** hydrogels, cardiac delivery, cardiac tissue engineering, bioprinting

## Abstract

Pathologies of the heart (e.g., ischemic disease, valve fibrosis and calcification, progressive myocardial fibrosis, heart failure, and arrhythmogenic disorders) stem from the irreversible deterioration of cardiac tissues, leading to severe clinical consequences. The limited regenerative capacity of the adult myocardium and the architectural complexity of the heart present major challenges for tissue engineering. However, recent advances in biomaterials and biofabrication techniques have opened new avenues for recreating functional cardiac tissues. Particularly relevant in this context is the integration of biomimetic design principles, such as structural anisotropy, mechanical and electrical responsiveness, and tissue-specific composition, into 3D bioprinting platforms. This review aims to provide a comprehensive overview of current approaches in cardiac bioprinting, with a focus on how structural and functional biomimicry can be achieved using advanced hydrogels, bioprinting techniques, and post-fabrication stimulation. By critically evaluating materials, methods, and applications such as patches, vasculature, valves, and chamber models, we define the state of the art and highlight opportunities for developing next-generation bioengineered cardiac constructs.

## 1. Introduction

The structure of the myocardial tissue is complex and finely tuned to support the continuous pumping function of the heart over a human lifetime (3 billion contraction cycles on average). Compared to other muscle apparatus, such as skeletal muscle (voluntary) or smooth muscle found in the intestine and arteries (involuntary), the myocardium is characterized by a unique cellular composition, extracellular matrix (ECM) organization, intrinsic anisotropy, and specific mechanical and electrical properties. These features arise from fundamental morphogenetic processes initiated during early embryonic development and completed postnatally through hypertrophic growth [1].

In recent years, two parallel advancements have laid the groundwork for cardiac tissue engineering. First, innovations in biomaterials and fabrication technologies have begun to address the challenge of replicating the myocardium’s structural complexity and functional anisotropy [2,3]. Second, the emergence of induced pluripotent stem cell (iPSC) technology has enabled the large-scale production of cardiomyocytes (CMs) for drug testing, in vitro modeling, and engineered grafts [4], leading to promising pilot studies in large animals and humans [5]. Despite this progress, several critical issues remain, particularly in achieving optimal electromechanical coupling between in vitro-generated and native tissues and ensuring the long-term stability of the engineered constructs.

**Figure 1:**
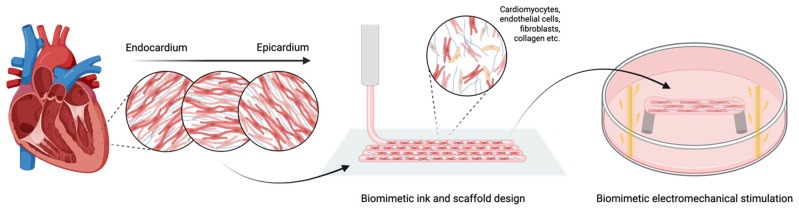
Integration of biomimetic ink, scaffold design, and physical stimulation to promote cardiac tissue maturation. Created in BioRender https://www.biorender.com/ (accessed on 18 July 2025).

This review aims to examine the strategies used to engineer cardiac tissues that replicate both the structure and function of the native heart. After outlining the key biological and mechanical features of cardiac tissue, an overview of main bioink materials and bioprinting techniques is provided. Emphasis is placed on the integration of biomimetic structural design and biophysical stimulation (mechanical and electrical) to promote tissue maturation (Figure 1). Finally, current applications in the fabrication of bioprinted cardiac patches, vascular structures, heart valves, and chamber-like models are evaluated, with a focus on integrating microscale and macroscale cardiac features to develop next-generation in vitro models and implantable constructs for advanced therapies.

## 2. Cardiac Tissue Features

### 2.1. Cardiac Development

Insights into the regulatory mechanisms of heart development can be harnessed to promote heart regeneration and repair or to design tissue engineering strategies. This is based on the concept that developmental paradigms are recapitulated during repair in postnatal tissue. The heart is the first organ that develops a function during early mammalian embryogenesis. Mouse and human heart development follow a comparable trajectory, with initiation occurring at gastrulation, around the end of the second week in humans (Carnegie Stage (CS) 7) and at embryonic day (E) 6.5 to 7.5 in mice [6]. The heart is derived from the mesoderm, the middle layer of the early trilaminar embryo germ layers originating from the primitive streak during gastrulation. The basic helix-loop-helix (bHLH) transcription factor MESP1 is the earliest protein detected in cardiac precursors, where the two mouse MESP paralogs are redundantly required for the specification of cardiac mesoderm [7]. The cardiac crescent emerges at E7.5 from the first population of cells to migrate to the heart-forming region, known as the first heart field (FHF). This migration of mesodermal cells is driven by MESP1-induced epithelial-to-mesenchymal transition (EMT) [8], and cardiogenic markers are first detected at this stage [9]. It has been shown that spontaneous asynchronous calcium oscillations occur in the forming cardiac crescent at E7.75 prior to the initiation of contraction at E8 [10].

The cardiac crescent subsequently expands and migrates to the midline fusing to form a linear heart tube. This is followed by a second wave of migrating cells, known as the second heart field (SHF), which induces the heart tube to undergo looping and elongation. The FHF is the region from where the prospective left ventricle, atrioventricular canal, sinus venosus and atria arise, whereas the SHF contributes to the outflow tract, the right ventricle and portions of the atria [8]. The SHF also gives rise to the proepicardial organ (PEO) which protrudes into the pericardial cavity and eventually forms the epicardial layer at E9.5 [11]. Heart tube looping (E8.5) occurs as a consequence of uneven growth and remodeling leading to the positioning of the venous pole anteriorly and the formation of primitive cardiac chambers. Once formed, the cardiac chambers undergo a complex maturation process involving septation and valve formation by E15, which corresponds to about eight weeks of development (CS22) in humans [6]. This allows the transition from a smooth heart tube containing immature endocardial and myocardial layers to generate the mature mammalian heart. It has been shown that cardiac neural crest cells, which migrate from the neural tube into the cardiac outflow tract, are not only required for septation [12], but also for normal heart tube looping [13].

The FHF and SHF lineages are regulated by complex regulatory networks involving components of the bone morphogenetic protein (BMP), sonic hedgehog (SHH), fibroblast growth factor (FGF), wingless-related integration site (WNT), and Notch pathways. For example, BMP arising from the adjacent endoderm induces CM specification, whereas WNT signals from the underlying neural tube and notochord repress it [14]. Both the FHF and SHF lineages are responsive to epicardial- and endocardial-derived signals such as retinoic acid and neuregulin, respectively [15]. Ultimately, a core set of evolutionary conserved transcription factors (NK2, MEF2, GATA, TBX, and HAND) is activated to control cardiac morphogenesis, cell fate, and the expression of genes encoding contractile proteins. These transcription factors also regulate each other to stabilize the cardiogenic program. Other cardiogenic factors that interact with the core set have also been described [9]. The upstream input signals activating the core cardiac transcriptional paradigm differ between the FHF and SHF. In the FHF, this is controlled by NKX2.5 (NK2 homeobox 5) and GATA4. However, it is driven by the LIM-homeodomain transcription factor ISL1, and the forkhead transcription factor FOXH1 in the SHF. It has been suggested that this is due to the evolutionary addition of the SHF which is unique to amniotes [16]. GATA4 and NKX2.5 are therefore central transcription factors in both the FHF and SHF [14].

Under the control of these transcriptional networks, cells committed to the CM lineage begin expressing sarcomeric proteins such as cardiac troponins and α-actinin, progressively assembling organized contractile units. Embryonic CMs are initially proliferative and display immature electrophysiological properties, including the absence of fully formed intercalated disks. As mammalian development proceeds, these cells irreversibly exit the cell cycle, undergo sarcomere maturation, and acquire adult-like structural and functional characteristics. Essential processes such as calcium handling, mitochondrial biogenesis, and gap junction formation are refined during late gestation, optimizing contractile function [17]. Additionally, CM lineage diversification into atrial, ventricular, and conduction system subtypes is tightly regulated by spatial and temporal cues, including retinoic acid gradients [18] and region-specific transcriptional programs such as TBX3 and IRX4 [19,20]. The precise orchestration of these events results in a fully functional, rhythmically contracting heart by mid-gestation.

Beyond CMs, the heart comprises various specialized cell types that originate from distinct embryonic progenitors. Endocardial cells, derived from the same mesodermal progenitors as CMs, line the heart interior and play essential roles in trabeculation, valve formation, and myocardial compaction. These processes involve bidirectional signaling with the myocardium mediated by pathways such as Notch, BMP, and Neuregulin-ErbB [21]. Epicardial cells undergo EMT to form epicardial-derived cells (EPDCs) that contribute to coronary smooth muscle cells, and cardiac fibroblasts (CFs) [22]. CFs, which regulate ECM and paracrine signaling, predominantly derive from EPDCs, with additional contributions from endocardial EMT [23]. This complex cellular crosstalk ensures the formation of a structurally sound and functionally competent heart.

Heart development is a highly conserved and tightly regulated process across metazoans, providing a basis for modeling congenital heart diseases in non-mammalian organisms. *Drosophila melanogaster* is an invertebrate that has been often employed as a representative model to investigate the core transcriptional machinery of cardiogenesis [24]. Its dorsal vessel, composed of contractile CMs and non-contractile pericardial cells, arises from bilateral cardiac progenitor cells (CPCs) that migrate and fuse at the midline, mirroring vertebrate cardiogenic fields [25]. Genes such as *tinman* (NKX2.5 ortholog) [26], *pannier* (GATA4) [27], and *neuromancer* (TBX20) [28] have shown conserved roles in cardiac cell fate specification. *Drosophila* models have been key to elucidating regulatory complexes like CCR4-NOT in cardiac gene expression and disease phenotypes, including dilated cardiomyopathy [29].

In zebrafish (*Danio rerio*), a two-chambered heart forms rapidly within 48 h post-fertilization [30]. The transparency of embryos and external development enables in vivo imaging of cardiac morphogenesis and cell migration. Zebrafish heart development and regeneration have been shown to share strikingly similar cellular and molecular mechanisms, reflecting the reactivation of developmental programs during tissue repair [31]. Both processes involve the proliferation and differentiation of CMs orchestrated by the core cardiac transcription factors. During heart development, CMs mature to form organized sarcomeres and establish functional contractile units, a process that is recapitulated during regeneration as surviving CMs dedifferentiate, re-enter the cell cycle, and rebuild the myocardial structure. Additionally, signaling pathways critical in embryonic heart formation, such as Notch, FGF, and Wnt, are re-engaged during regeneration to coordinate CM renewal [30]. Both contexts also feature dynamic remodeling of the ECM and involvement of EPDCs, which contribute to new vasculature and fibroblast populations essential for restoring heart function [32,33]. This shared reliance on conserved transcriptional networks and signaling cascades underscores how zebrafish regeneration harnesses developmental biology programs to achieve efficient cardiac repair. Collectively, research model systems provide a mechanistic framework for understanding human heart development and regeneration.

### 2.2. The Evolving Structuring of Cardiac Matrix

In its definitive shape, the left and right cardiac chambers are structurally and electrophysiologically programmed to control blood flow with well-coordinated axial movements [34], involving clockwise and counterclockwise torsional motion to attain optimal blood filling and ejection [35]. While we refer to specific reviews and textbooks for an in-depth description of the myocardial tissue structure, it will be sufficient here to recall that the tissue is highly anisotropic and has specific mechanical characteristics that are established during a process of mechanical maturation that occurs during the transition from the embryonic to fetal stage, and from the fetal to adult stage [36]. This is important for tissue engineering strategies that have as a principal aim the recreation of geometrically, as well as mechanically, compliant scaffolds [2]. The composition, the architecture and the mechanical characteristics of the ECM are particularly interesting in this scenario. In fact, the cardiac ECM is deposited by CFs from the earliest stages of cardiogenesis during the embryonic and fetal life, to provide structural integrity and mechanical support to the growing and definitive contractile cells. This maturation process has been the subject of studies in which it was clarified that the evolving structure and mechanics of the cardiac matrix reflect a specific maturation program involving not only changes in composition but also matrix stiffening, to instruct proper differentiation of the contractile cells [37]. The major proteins in ECM are: collagen type I and collagen type III which compose the interstitial and the perivascular matrix [38], collagen type I, II, III, and V that are specifically needed to preserve the mechanical integrity and collagen type VI that maintains tissue structure. The alignment of collagen fibers is highly anisotropic, following a deposition pattern dictated by the distribution of the uniaxial forces that control cardiac beating [39]. Laminin and fibronectin play a role in promoting cell adhesion, spreading, and migration, while glycosaminoglycans (GAGs), like heparin, enhance heart viscoelastic properties and support anchoring of essential growth factors [40]. With such a precise composition and nanostructural features, researchers face challenges in recapitulating the ECM in cardiac tissue engineering through design and fabrication procedures adapted to the complexity of the myocardium.

## 3. Bioink Materials

Bioinks can be generally defined as cell-containing formulations that are compatible with automated biofabrication technologies [41]. For cardiac applications, most bioinks are scaffold-based, meaning that cells are encapsulated within biomaterials, usually high-water-content formulations of polymers or hydrogel precursors enriched with bioactive molecules.

Bioink materials must be biocompatible and printable, while also meeting the mechanical and structural requirements of the construct. Post-printing, they should provide cells with proper biomimetic cues to maintain viability and support migration, proliferation, differentiation and maturation, ultimately creating a microenvironment that promotes the development of tissue-level functions. Finally, the scaffold materials should ideally degrade over time and be replaced by ECM proteins secreted by the embedded cells [42].

From the perspective of mimicking native ECM, natural biomaterials composed of proteins, polysaccharides, and/or GAGs are highly preferred.

Collagen stands out as the primary ECM protein in cardiac tissue, playing a crucial role in cellular growth, organization, and signaling. Its thermoresponsive properties allow for sol-to-gel transition at physiological temperatures, making it ideal for cell encapsulation [43]. Gelatin, derived from the partial hydrolysis of collagen, shares structural similarity with ECM proteins and offers excellent biocompatibility. It also exhibits thermal gelation, forming hydrogels below 30 °C (for mammalian gelatin) [44]. Its lower cost and greater availability compared to collagen make it an appealing candidate for bioink formulation in cardiac tissue engineering. However, the mechanical properties of both collagen and gelatin are weak due to their low viscosity. Their physical crosslinking through thermal or pH-driven processes, which relies on weak, non-covalent interactions, results in hydrogels suitable primarily for temporary structures or sacrificial materials [45,46]. To address this limitation, strategies such as blending collagen and gelatin with other polymers like alginate [3,47,48], hyaluronic acid [49] and fibrin [50,51] have been employed to enhance their stability and rheological properties. Additionally, chemical modification into methacrylated derivatives, such as methacrylated collagen (MeCol) [52] and methacrylated gelatin (GelMA) [53,54], enables photocrosslinking, resulting in semi-synthetic bioinks that combine the biological benefits of natural polymers with the tunable mechanical properties of synthetic modifications.

Fibrinogen, a plasma glycoprotein, is enzymatically cleaved by thrombin to form fibrin, a fibrous hydrogel that includes integrin-binding sequences promoting cell adhesion and endothelialization. Fibrin’s bioactivity stems from its ability to concentrate and retain growth factors, such vascular endothelial growth factor (VEGF), which aid angiogenesis and vascularization, key benefits for cardiac constructs [55]. Fibrin gels exhibit excellent biocompatibility, rapid gelation, and quick degradation, which can be regulated by incorporating protease inhibitors in the gel formulation or in the culture medium [56]. However, fibrin’s low viscosity and weak mechanics pose challenges for standalone use and necessitate blending with materials like gelatin [50,57], alginate [58], and hyaluronic acid [59] to enhance stability and structural integrity.

Alginate is a natural anionic polysaccharide primarily derived from the cell walls of brown algae. It crosslinks ionically in the presence of divalent cations like calcium, with gelation rates varying based on the crosslinker’s solubility—for example, rapid gelation with calcium chloride (CaCl_2_) or slower, more uniform gelation with calcium carbonate (CaCO_3_). A double-step crosslinking process (internal pre-printing with CaCO_3_ and external post-printing with CaCl_2_) can enhance shape fidelity and long-term structural stability [60]. Optimizing the total crosslinking time is crucial, as it significantly impacts cell viability. The mechanical properties and viscosity of alginate can be tailored by altering its molecular weight, concentration, and the type and concentration of the crosslinker used [61,62]. This versatility, combined with its high biocompatibility, makes alginate a widely used bioink component. However, its bioinert nature limits cell adhesion and proliferation, necessitating modifications such as the addition of bioactive peptides like RGD sequences [63] or blending with cell-friendly materials like gelatin [48] and silk fibroin [64]. While mammals cannot naturally degrade alginate due to the absence of specific lyase enzymes, ionically crosslinked alginate gels can dissolve as the divalent ions maintaining the gel structure are replaced by monovalent cations from the surrounding medium. Additionally, the partial oxidation of alginate, which introduces reactive groups accelerating breakdown, can enhance its degradation rate under physiological conditions [65].

Heart-derived decellularized extracellular matrix (dECM) is a standout natural material for cardiac bioinks due to its complex composition, which includes proteins, GAGs, and growth factors. Compared to simpler bioinks based on single proteins, dECM provides a more biomimetic environment that better supports tissue-level functions. The preparation of dECM involves treating cardiac tissue, typically sourced from porcine or ovine hearts, with detergents to remove cellular components while maintaining the extracellular structure. This is followed by lyophilization and enzymatic digestion with pepsin. Once solubilized, dECM can undergo thermal gelation at physiological temperatures, forming hydrogels suitable for three-dimensional (3D) bioprinting [66,67]. Despite its advantages, dECM presents challenges due to its high concentration requirement for printing, as well as its fibrous, low-modulus nature, which affect scalability, structural fidelity, and printability. To address these issues, methods like dual crosslinking, combining thermal gelation with photo-polymerization [68,69], and the incorporation of methacrylated polymers such as GelMA [70,71,72] have been employed. Additionally, the incorporation of photopolymerizable PEG-DA and Laponite has allowed for the creation of constructs with adjustable elasticity, enabling the replication of both healthy and fibrotic cardiac tissue stiffness [73].

Despite their superior mechanical properties, the use of synthetic biomaterials does not represent the preferred choice for biomimetic approaches, due to the lack of active binding sites essential for cellular interactions. In this context, they are primarily used as supportive inks to create polymeric frameworks (e.g., polycaprolactone (PCL) [59,68,69]) or as sacrificial polymers (e.g., Pluronic F-127 [51,74]).

## 4. Bioprinting Techniques

Some of the main categories of bioprinting methods employed in the field of cardiac tissue engineering are represented by extrusion-based bioprinting, embedded bioprinting, inkjet-based bioprinting, laser-assisted bioprinting, stereolithography and digital light processing (Table 1).

### 4.1. Extrusion-Based Bioprinting and Embedded Bioprinting

Extrusion-based bioprinting (EBB) is the most applied technique in cardiac bioprinting applications. The bioink, loaded into a syringe, is extruded through a nozzle as continuous strands to form 3D structures in a layer-by-layer manner. The extrusion mechanism relies on a constant pressure that can be generated by either a pneumatic or a mechanical (piston- or screw-based) system [75,76]. The main parameters affecting the printing process are the nozzle speed, the applied pressure, and the nozzle diameter [77].

EBB allows the selection of a wide variety of materials, spanning from low-viscosity gels to highly viscous polymers (up to 6 × 10^7^ mPa·s). Additionally, most up-to-date extrusion-based bioprinters are equipped with multiple extruders, which enable the simultaneous use of different biomaterials and cell types and therefore the creation of complex, heterogeneous structures. This technique is also particularly effective for the biofabrication of tissues with high cell density and is relatively cost-effective, making it accessible and widely adopted [66,75,78].

However, EBB presents some significant disadvantages. The resolution of the printing is generally lower compared to other techniques (~100 μm), limiting the precision with which cells can be organized within the structures. Moreover, the mechanical forces exerted during extrusion can compromise cell viability, especially when using small-diameter nozzles or high pressures [79].

Conventional EBB also allows for the creation of large and complex designs, but it typically requires the use of scaffolding structures. An alternative form of EBB, named embedding bioprinting, overcomes this constraint by extruding bioinks in supporting baths with proper rheological or mechanical properties to temporarily support soft or overhanging structures, exploiting a gel-in-gel approach [80,81].

### 4.2. Inkjet-Based Bioprinting

The use of inkjet-based bioprinting (IBB) for cardiac tissue engineering applications is still at an early stage. It involves the precise deposition of bioink droplets onto a collector plate [82]. This is achieved through either thermal or piezoelectric mechanisms embedded in the printhead. In thermal IBB, a heater is used to generate small air bubbles that create pressure pulses to eject the bioink, while piezoelectric IBB employs a piezoelectric actuator to generate the force needed to expel the bioink droplets [83,84].

One of the significant advantages of IBB is its high resolution (<100 μm), since it can generate droplets as small as 1–100 pL, enabling the creation of fine patterns [85]. Additionally, the technique allows for the formation of concentration gradients by varying droplet size and density during the printing process, which could be particularly useful in tissue engineering applications where different cell and molecule densities are required across a construct [86]. IBB is a low-cost method characterized by a relatively rapid printing speed and high cell viability [87,88].

Nevertheless, IBB has considerable limitations. The technique requires bioinks with low viscosity (<20 mPa·s) [89], which can restrict the mechanical strength and structural integrity of the printed constructs. This limitation is particularly problematic when trying to fabricate tissues that need to support higher loads or stresses, such as cardiac tissues. Furthermore, the low viscosity requirement limits the density of cells that can be encapsulated in the bioink, which can affect the biological functionality of the printed tissue. Nozzle clogging is also a frequent issue [90].

### 4.3. Laser-Assisted Bioprinting

Laser-assisted bioprinting (LAB) leverages high-intensity laser pulses to deposit bioink droplets in a precise, non-contact manner. A LAB system typically consists of three main components: a pulsed laser beam, a donor “ribbon”, and a collector substrate. The ribbon is composed of a transparent support slide coated with an energy-absorbing metal layer and a cell-containing bioink layer. When the laser beam is focused onto the ribbon, the generated energy is absorbed by the metal, creating a vapor bubble that propels a droplet of bioink onto the receiving substrate.

The non-contact, nozzle-free nature of LAB technology ensures high cell viability by minimizing mechanical stress during printing and eliminates the nozzle clogging issues commonly found in other bioprinting methods. By carefully tuning factors such as laser fluence, bioink viscosity, and bioink thickness, the LAB technique can achieve high resolution. Moreover, LAB is compatible with high cell densities and a considerable range of bioink viscosities (1–300 mPa·s) [42,91,92].

However, LAB is not without its drawbacks. The technique is complex, requiring precise control over laser parameters such as fluence, intensity, and pulse duration. This complexity, along with the high cost of the laser systems, makes LAB less accessible compared to other bioprinting methods. Additionally, while LAB excels in creating 2D and thin 3D constructs, it is often limited in its ability to fabricate thicker, multilayered structures. The small scale of the printed structures and the long preparation time required for each ribbon limit its application, particularly in clinical settings [42,86].

### 4.4. Stereolithography and Digital Light Processing

Stereolithography (SLA) and digital light processing (DLP) are two prominent light-based bioprinting techniques that leverage photopolymerization to create 3D structures. Both methods rely on light, typically in the UV spectrum, to selectively solidify photosensitive bioinks layer by layer, but they differ in their light source and approach.

An SLA system consists of a laser light source, a liquid bioink tank, and a movable build stage. In a bottom-up setup, the laser beam is directed at the bottom of the resin vat from below, tracing the pattern of a cross-section of the part spot-by-spot. After the entire layer is cured, the build platform moves upwards, making the part gradually emerge from the liquid. While similar to SLA in principle, DLP employs a digital micromirror device to project images of complete layers onto the resin, polymerizing a whole layer in a single exposure phase [93,94].

SLA typically offers higher resolution with more complex laser control, while DLP excels in speed and simplicity since it does not require beam scanning. Both techniques are nozzle-free and thus avoid nozzle clogging and do not introduce shear stress to cells.

However, the use of UV light and photoinitiators can negatively impact cell viability by causing cytotoxicity and damaging DNA. Researchers are addressing this issue by exploring visible light-based or photoinitiator-free photopolymerization methods [95,96]. Additionally, cell sedimentation can occur during SLA or DLP bioprinting, especially in thicker scaffolds, leading to inhomogeneous cell distribution [78].

**Table 1:** Main advantages and limitations of 3D bioprinting techniques.

Techniques	Main Advantages	Main Limitations	Ref.
EBB	Compatible with a wide range of material viscosities Enables multi-material printing Supports high cell densities Accessible and widely adopted	Low resolution (~100 µm) Shear stress during extrusion may reduce cell viability	[66,75,78,79]
IBB	High resolution (<100 µm) Fast printing speed High cell viability Low-cost setup	Requires low viscosity bioinks Limited cell densities Prone to nozzle clogging	[85,86,87,88,89,90]
LAB	Nozzle-free technique High cell viability High resolution Compatible with high cell densities and a broad range of bioink viscosities	High equipment cost Complex setup and parameter tuning Limited to small-scale or thin-layer constructs	[42,86,91,92]
SLA and DLP	High resolution (especially SLA) Fast printing speed (especially DLP) Nozzle-free techniques	Potential cytotoxicity from UV light and photoinitiators Risk of cell sedimentation and uneven cell distribution	[95,96]

## 5. Biomimetic Structural Design

The organization of cardiac tissue is essential for the execution of its mechanical and electric function. Native myocardium is composed of aligned myofibers that exhibit a gradual transition in their orientation throughout the myocardial wall layers. In the left ventricle it varies from a right-handed helical arrangement in the innermost layer (subendocardial layer) to a left-handed helical arrangement in the outermost one (subepicardial layer) [97,98]. At the tissue level, each layer is made up of an anisotropic array of cardiac cells and ECM fibers packed together to coordinate the contraction of the whole ventricle.

The understanding of the structural alignment effect on various cardiac tissue mechanisms is vital for a wide range of applications, spanning from in vitro disease modeling to in vivo heart repair [99]. To effectively mimic this complex 3D anisotropic structure, different tissue engineering methods have attempted to develop scaffolds that can guide the orientation of seeded cardiac cells thanks to nano- and micro-scale topographical features. Examples include aligned nanofibers formed via electrospinning [100,101], substrates patterned with proteins via microcontact printing [102], and substrates with contact guidance cues such as microgrooves [103,104], microlattices [105,106], and microchannels [107]. However, most of these approaches can only induce cellular alignment in 2D and rely on seeding cells onto previously fabricated scaffolds, without the possibility to precisely control their spatial distribution [54].

**Figure 2:**
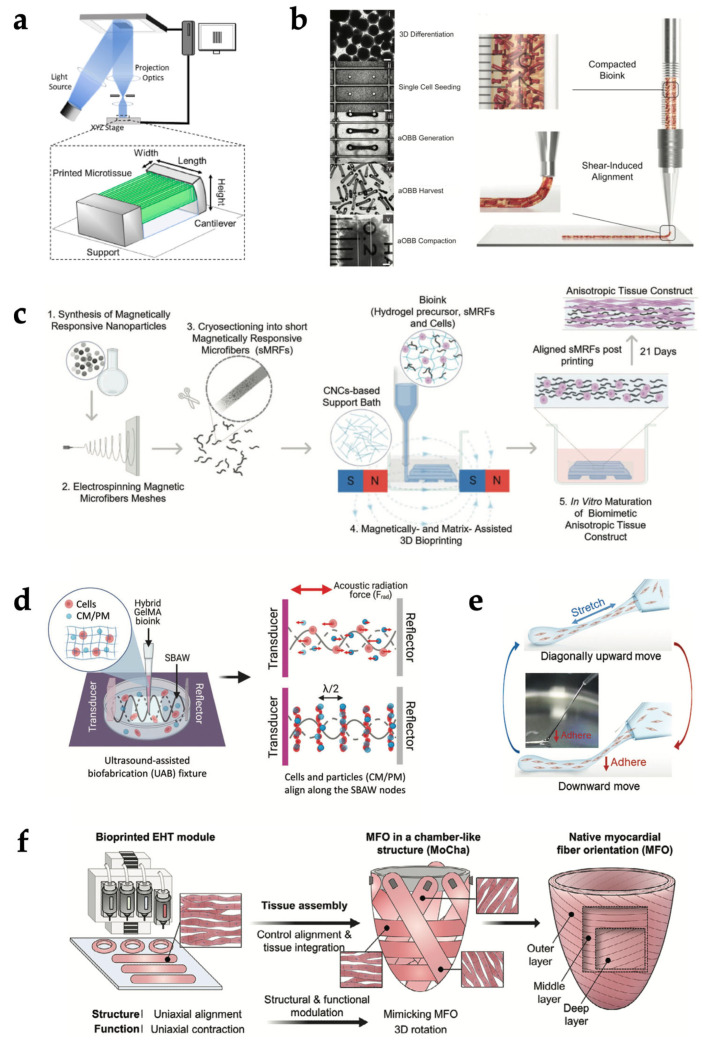
Bioprinting strategies for structural biomimicry of cardiac tissue. (**a**) Schematic illustration of parallel-line pattern printing via a microscale continuous optical printing system. Adapted with permission from [54]. © 2020, Elsevier. (**b**) Image sequence showing aOBB bioink preparation (left) and schematic illustration of aOBB shear-induced alignment during extrusion (right). Adapted with permission from [50]. © 2022, Wiley-VCH GmbH. (**c**) Schematic illustration of magnetic field-assisted bioprinting to align magnetically responsive microfibers within the bioink. Adapted with permission from [115]. © 2022, Wiley-VCH GmbH. (**d**) Schematic illustration of cells and particles patterning via ultrasound-assisted biofabrication. Adapted with permission from [116]. © 2022, The Authors. Advanced Healthcare Materials published by Wiley-VCH GmbH. (**e**) Schematic illustration of “sewing-like” fabrication method relying on static stretching. Adapted with permission from [117]. © 2022, Wiley-VCH GmbH. (**f**) Schematic diagram of bioprinting-assisted tissue assembly to reproduce myocardial fiber orientations in a chamber-like structures. Adapted with permission from [121]. © 2024, The Authors. Advanced Materials published by Wiley-VCH GmbH.

3D bioprinting represents an excellent platform to arrange cell-laden bioinks in a layer-by-layer fashion, to fabricate native cardiac tissue-like architectures with anatomical precision over different length scales (Figure 2). The mild fabrication temperature and the ease of integrating multiple biomaterials make bioprinting particularly suitable for developing anisotropic living constructs with high cellular density [108].

The choice of a bioprinted geometry consisting of 3D parallel strands [54,59,71,109], or a grid with varying fiber spacing across successive layers [110,111,112], represents a first option for mimicking cardiac native anisotropy at the macroscale.

Wang et al. [59] used EBB to print CM-laden filaments made of a fibrin-based composite bioink, alternating with strings of a sacrificial hydrogel, both anchored to a PCL frame. The anchoring points allowed intrinsic forces to induce a compaction phenomenon that facilitated the development of muscle fibers and, together with the aligned architecture generated by 3D printing, synergistically promoted CM orientation and maturation. Liu et al. [54] encapsulated neonatal mouse CMs in 3D GelMA parallel lines, patterned via microscale continuous optical printing (based on DLP technology), and tensioned between a support and a flexible cantilever (Figure 2a). CMs encapsulated within the 3D lines showed greater sarcomere alignment, cellular compaction and produced a twofold higher contractile force compared to CMs seeded atop both 2D samples and 3D lines. In contrast, patterning cells in structures with isotropic features (e.g., slab or grid) disrupted the ability of CMs to align and contract along the main axis of the scaffold, significantly reducing the contractile force.

Zhang et al. [110] engineered a micro-fibrous scaffold composed of alginate and GelMA, with encapsulated HUVECs and neonatal rat CMs distributed on the endothelialized microfibers. An increase in the aspect ratio of the unit grid led to higher connexin 43 protein levels, improved CM alignment along the direction of the microfibers, and greater amplitude of spontaneous contractions. Greater macroscale anisotropy correlated with longer spontaneous beating durations and a reduced decline in beating frequency over time. Mehrotra et al. [111] developed cardiac patches with a similar grid pattern by extruding a silk-based bioink composed of non-mulberry silk fibroin, polyethylene glycol di-methacrylate, and GelMA. Neonatal rat CMs cultured on these scaffolds exhibited excellent attachment and growth, along with high levels of sarcomeric α-actinin and connexin 43 proteins, as well as increased expression of troponin T (*Tnni3*) and sarcomeric actinin (*Actn1*) genes, indicating maturation toward an adult phenotype.

An alternative strategy for engineering anisotropy exploits forces of various kinds to induce the alignment of micro- or nanosized elongated bioink components or directly of cells along precise directions. For instance, wall shear forces generated in the tapered nozzle during EBB can be leveraged for this aim [108,113]. Choi et al. [3] developed 3D organ-level scaffolds using a gelatin-alginate ink containing gelatin fibers prefabricated via rotary jet spinning [2]. The shear-induced alignment of fibers during printing provided microscale cues that mimic the native ECM and promote the self-organization of seeded human CMs into anisotropic muscular tissues in vitro, thereby allowing the simultaneous recapitulation of microstructural architecture and macrostructural chamber geometry. In a recent study, Ahrens et al. [50] extruded densely cellular and anisotropic organ building blocks (aOBBs)—resulting from the self-assembly of human iPSCs-derived CMs (hiPSC-CMs) and fibroblasts into cardiac microtissues on a pillar platform—within a gelatin-fibrinogen mixture (Figure 2b). These aOBBs aligned along the print path due to the same flow-induced and extensional forces that can orient acellular fibers.

It is well known that greater shear stresses determine an improvement in the alignment of high-aspect ratio elements, but they can also irreversibly damage cells suspended in the bioink. Therefore, flow-induced forces must be carefully balanced to optimize both alignment and cell viability, by tuning printing parameters such as nozzle diameter, extrusion pressure, and ink viscosity [108]. To overcome the limitations of shear-based alignment, researchers have investigated the application of external electric, magnetic, or acoustic fields post-printing to disrupt the homogeneous distribution of bioink elements and arrange fibers and cells. Kim et al. [114] included golden nanowires in a collagen-based bioink to provide aligned topographical cues to the laden myoblasts. Nanowire alignment was achieved through extrusion via a micro-sized nozzle and the subsequent application of an electric field (5 V, 1 Hz), incubating the construct between two indium tin oxide electrodes. Results showed that the aligned nanowires created an effective asymmetric electrical microenvironment, which promoted actin fiber orientation and significantly increased the expression of myosin heavy chains compared to control constructs without electric field stimulation. Furthermore, constructs with electrically aligned golden nanowires enhanced muscle tissue regeneration following implantation. Pardo et al. [115] demonstrated a magnetically and matrix-assisted bioprinting method using short magnetically responsive microfibers (sMRFs) (Figure 2c). They were realized cryosectioning an electrospun mesh made of PCL and zinc-doped magnetic nanoparticles. Under a weak magnetostatic field (14 ± 2 mT) provided by a two parallel magnet system, microfiber alignment was achieved without compromising the structural resolution. In hydrogels containing high concentrations of aligned sMRFs (2.00 mg mL^−1^), encapsulated human adipose-derived MSCs exhibited significantly increased aspect ratios and pronounced alignment along the fiber axis after 7 days. Increasing fiber concentration decreased inter-fiber spacing and maximized the contact guidance phenomenon between cells and fibers. Notably, cell orientation was maintained even in curved regions of the printed pattern, and constructs preserved this alignment over extended culture periods. In another study by Chansoria et al. [116], a versatile ultrasound-assisted biofabrication technique was hybridized with EBB to fabricate constructs featuring 3D perfusable channels for vasculature and a crisscross organization of cells and bioadditives (collagen microaggregates or PCL microfibers). Under the action of the acoustic radiation forces, cells and particles aligned along the nearest pressure node of the bulk acoustic wave (Figure 2d). When patterned alongside the cells, the bioadditives guided cell alignment and ECM deposition along the structured arrays, resulting in biomimetic structural and mechanical anisotropy.

Since some recent studies reported that static stretching can also induce cellular alignment, He et al. [117] developed a “sewing-like” method to control the cellular orientation of patterned cellular constructs (Figure 2e). It is based on the extrusion of a cylindrical filament made of a hydrogel-cell mixture (acting as a “sewing thread”) from a Teflon tube, the subsequent adhesion of its leading end to a Petri dish substrate, and the movement of the tube during extrusion diagonally upward and then vertically downward, stretching the filament and then making it adhere to the dish piece by piece. The persistent presence of static tensile stress even after the filament adhesion was deemed to be the sole cause of the elongation and the division of cells in the direction of stretching. Moreover, the authors of the study suggested the possibility to adapt this process to EBB.

Overall, in an anisotropic organization, the most common consequence of cell alignment is that cardiac cells become elongated, with more aligned actin filaments, myofibrils, and Z-lines, as well as higher levels of proteins involved in both contractile and conductive properties, such as sarcomeric α-actinin and connexin 43. Enforcing an elongated shape on cardiomyocytes also enhances their contractile force and promotes maturation in terms of electromechanical coupling, resulting in a more adult-like phenotype [98,111].

To build more complex anatomical models, the design of multilayer scaffolds with layer-specific control of cellular orientation and organization would be advantageous, although it remains a significant challenge. Various research groups have achieved multiaxial arrangements by stacking [100,118,119] or wrapping [120] aligned cellular structures. Wu et al. [118] constructed a two-layered nanofiber yarn network by combining electrospinning with a weaving method. Eom et al. [119] and Liu et al. [100] performed layer-by-layer modular assembly of three unit scaffolds stacked at different angles, fabricated via hollow patterned electrolyte-assisted electrospinning and through the integration of 3D printing with electrospinning, respectively. Mohammadi et al. [120] wrapped three 2D aligned trapezoidal cell sheets with different orientations around a central mandrel to obtain a functional, conically shaped chamber model. However, these hierarchical approaches still face limitations in terms of scalability, which is required to faithfully replicate organ-level geometry.

Lately, a larger ventricle model incorporating circumferential and helical alignment was developed using rotary jet spinning technology [2]. Although this strategy generated a chambered fiber structure mimicking native ECM architecture, cells were primarily distributed on the surface and not in a transmural manner, with limited CM penetration into the scaffolds and a lack of synchronization between layers, which is essential for reproducing the structure-function relationship of the left ventricle.

Thus, in an even more recent work, Hwang et al. [121] proposed the synergistic integration of bioprinting and tissue assembly technologies to fabricate a heart analog with three-layered right-handed helical, circumferential, and left-handed helical orientations (Figure 2f). Firstly, uniaxially aligned engineered heart tissue (EHT) modules were manufactured using a multicellular bioink and reproduced both the structural and functional (i.e., contractile and electrophysiological) features of myocardial fibers. These building blocks were assembled on a customized EHT assembly platform, designed in two distinct parts: one for guiding EHTs to the desired angles and the other for managing the chamber-like geometry. The second part was removed after the tissue assembly to observe the resulting between-layer coupling, enabling free-standing left ventricle-like motions of the construct, such as multiaxial deformation and ventricle twist. However, manual assembly poses a limitation, as it can lead to variability in alignment precision and reduce reproducibility.

The concept of pre-patterning cells in specific geometries may be of significant interest also in developing cardiac pathological models, where disease phenotypes characterized by myocyte disarray need to be recapitulated. To this end, structures with more isotropic features should be investigated [54]. Future disease models should address the dynamics of reorganization to understand how the myocardium reorganizes under pathological conditions and whether this disorganization is reversible [98].

## 6. Biomimetic Physical Stimulation

The human heart is a dynamic electromechanical system, with the myocardial tissue subjected to cyclic physical stresses from very early development, without pause, for a person’s entire life [122]. As the contraction of CMs in vivo is initiated and synchronized by the electrical excitation originating in the sinus node, cardiac cells are exposed to a pulsatile electrical field with an amplitude of 0.1–10 V/cm and pulse duration of 1–2 ms, typically at a rate of 60–100 beats per minute (bpm) [123,124]. At the same rate, myocardial structure experiences both active stretching during filling and self-generated mechanical force during ejection. In a healthy human heart, the ranges of pressure developing during one cardiac cycle (10–120 mmHg for the left ventricle, 5–30 mmHg for the right ventricle [125]) are transduced as local mechanical stresses and can reach up to 50 kPa, with mean strains of 22.9% longitudinally and 59.2% radially [126]. The resulting combined physical cues influence myocardial homeostasis and pathophysiology. The development and maturation of CMs are driven by the balance between extrinsic and intrinsic physical loads that regulate protein synthesis, sarcomere assembly, cell size, contractile activity, interactions with other cells, and remodeling of the ECM [127,128]. In turn, the continuous remodeling of the ECM influences the transfer of loads to the cells, in a positive feedback mechanism that helps maintain the homeostasis of the heart [129,130]. However, this dynamic reciprocity between cells and ECM can also be responsible for the development of pathologies, as in the case of cardiac fibrosis, in which deranged electromechanical stimuli activate CFs, inducing their differentiation into collagen-producing pro-fibrotic cells, myofibroblasts [131,132,133]. The activated self-sustaining positive feedback can evolve into a chronic response, with subsequent enhanced ECM deposition and stiffening, ultimately resulting in cardiac dysfunction [134,135,136,137,138]. At the molecular level, the translation of mechanical forces into biochemical signals (a process known as mechanotransduction) is mediated by key signaling pathways. In this context, of particular relevance is the Yes-associated protein (YAP) and its paralog TAZ, transcriptional regulators whose nuclear localization and activity are tightly controlled by cytoskeletal tension and substrate stiffness. Persistent YAP/TAZ activation has been directly implicated in fibroblast-to-myofibroblast transition and cardiac fibrosis [132,138,139,140]. The integration of mechanosensitive cascades ultimately determines cellular fate and tissue-level outcomes, underscoring the necessity of including mechanical stimulation as a critical parameter in the maturation and functional validation of bioengineered cardiac constructs.

The inner complexity of native cardiac tissue makes the fulfillment of reliable functional in vitro cardiac models highly challenging, as the cultured myocardial tissue should propagate electrical impulses and respond by synchronized contractions. To achieve these conditions, CMs, which are highly sensitive cells that quickly dedifferentiate in the absence of physical cues [141], must mature into an interconnected syncytium [142]. For this reason, over the last 20 years, cardiac tissue engineering research has developed and adopted dynamic culture devices (bioreactors) to mimic the native cardiac environment and its physical cues [143,144,145,146]. Several groups have demonstrated that providing in vitro the most relevant physical stimuli of the myocardium, such as cyclic stretch and/or electrical pulses, is fundamental for generating functional substitutes for myocardial tissue [147,148,149,150,151,152,153,154,155,156,157,158].

In particular, customized bioreactors and commercial devices have been developed and used to impose cyclic stretch on flexible substrates or 3D constructs, leading to enhanced cell proliferation, myocardium-like organization, and increased contractile performance of engineered cardiac tissues [159,160,161,162,163]. In parallel, setups for delivering electric field stimulation have flourished, allowing researchers to demonstrate the positive effects of monophasic or biphasic pulses on the rate, duration, and number of action potentials in CMs, with enhanced cell–cell coupling and calcium handling, and increased maturation of the stimulated cardiac constructs [164,165,166,167,168,169,170,171,172].

In this section, an overview of the most significant efforts to develop biomimetic culture platforms delivering controlled biophysical stimuli to 3D biofabricated constructs and the outcomes obtained are presented.

### 6.1. Mechanical Stimulation

Due to the mechanical properties of hydrogels, mechanical forces have been generally applied through two main methods: passive auxotonic loading and active cyclic loading.

Passive auxotonic loading is typically achieved by suspending the construct between flexible posts, which generate a passive force in response to the combination of hydrogel shrinkage and CM contraction [173].

To obtain hydrogel constructs embedding flexible posts, the most common strategy is to polymerize the construct into a mold where the posts are positioned from above [174]. Exploiting this principle, Hansen and colleagues in 2010 developed fibrin-based constructs containing rat cardiac cells, suspended on flexible silicone posts [175]. By monitoring the contractile activity with a camera, they observed rhythmic deflection of the posts after 8 days of culture. Moreover, the constructs exhibited longitudinally aligned actinin-positive cardiac muscle networks and lectin-positive vascular structures homogeneously interspersed throughout the construct. In 2018, the effects of static auxotonic loading applied to collagen I hydrogels seeded with hiPSC-CMs at three different initial lengths were monitored for up to 50 days and coupled with computational modeling, providing a predictive model for optimizing cell alignment and calcium dynamics within the constructs [176].

The flexible post approach has also been refined by exploring the possibility of modifying post geometry or structure. Using two different configurations, neonatal mouse CMs and fibroblasts suspended in a collagen—Matrigel hydrogel were subjected to uniaxial and biaxial constraints, mimicking ‘healthy’ aligned and ‘diseased’ disorganized cardiac matrices [177]. Matrix misalignment led to a stellate cell shape and disorganized sarcomere organization, while CMs in aligned matrices were more elongated and had aligned sarcomeres. Structural modifications of the posts have been recently investigated, including a bioreactor capable of providing auxotonic loading via two metallic wires: one flexible, made of Nitinol, and the other rigid, made of steel [178]. Using fibrin hydrogel constructs containing a mixture of CMs and fibroblast, the researchers observed the formation of bizonal tissues with differential maturation. CMs exposed to high passive loading exhibited a rounded morphology and poor contractility, while those exposed to low passive mechanical stimulation showed enhanced maturation and organization.

Beyond post-based approaches, an alternative strategy was proposed by Lui and colleagues in 2021 [179]. Using a 3D bioprinter, scaffold-free cardiac tissue grafts were produced by assembling hiPSC-CMs cell spheroids into arrays (4 mm × 2 mm × 0.8 mm) held in place by stainless steel needles. After spheroid fusion, the tissue constructs were subjected to static loading using PDMS molds. Static mechanical stretching increased sarcomere length, maximal contractile force, and ECM alignment in the constructs, while decreasing their elastic modulus.

Differently, active cyclic loading is achieved by developing dedicated automated mechanical setups based on a motor, which can hold or grasp the distal portions of the constructs and impose a specific waveform controlling the force or the displacement.

In this regard, one of the first setups to achieve functional improvement of biofabricated cardiac tissues with stretch was developed by the Eschenhagen group and consisted of two rigid metallic posts placed horizontally inside a Petri dish and externally actuated [147]. Ring-shaped constructs made of collagen I hydrogel and rat cardiac cells were subjected to unidirectional cyclic stretch (10% strain, 2 Hz), resulting in the development of a construct characterized by longitudinally oriented cell bundles, with morphological features of adult tissue [180]. Although the contractile force was still much lower than that of the native tissue, the subsequent refinement of the model led to some impressive results, such as the successful implantation of the constructs in infarcted rat hearts [181]. Tulloch and colleagues in 2011 modified a Flexcell FX-4000T bioreactor (Flexcell International Corp., Hillsborough, NC, USA) by attaching nylon mesh tabs to the deformable silicon floor of the wells, obtaining a system able to transmit cyclic uniaxial tension to the constructs [182]. With this system, collagen I hydrogels containing CMs mixed with HUVECs and marrow stromal cells were subjected to uniaxial mechanical stress and exhibited enhanced matrix fiber alignment, myofibrillogenesis and sarcomeric banding, as well as increased CM hypertrophy and proliferation. More recently, an automated bioreactor platform that can apply tunable cyclic stretch to engineered cardiac tissue constructs of different shapes, dimensions and mechanical properties and monitor their mechanical response in situ was developed [183]. The system was first used to culture engineered cardiac constructs based on decellularized human skin scaffolds seeded with human CPCs, obtaining increased cell migration towards the inner layers of the scaffolds with mechanical stimulation, as well as increased tissue maturation [184]. Then, using 3D annular cardiac tissue models composed of neonatal rat cardiac cells embedded in fibrin hydrogel, researchers demonstrated that cyclic stretch (10% strain, 1 Hz) significantly enhances CM alignment, maturation, and contractility compared to static conditions. In situ monitoring revealed increasing passive force during dynamic culture, indicating maturation progression. Notably, only stretched constructs exhibited responsive and synchronized contractile activity under external electrical pacing. Finally, in 2024, researchers presented a platform directly integrating bioprinting with a bioreactor designed to mechanically stimulate 3D skeletal muscle constructs [185]. The tissue model was based on a commercial bioink (CELLINK, Göteborg, Sweden) composed of nanofibrillated cellulose, alginate, and fibrinogen, ionically crosslinked with a diluted calcium chloride solution, mixed with murine myoblasts. The construct was then bioprinted directly onto stretchable PDMS supports, embedded in a modular culture chamber, for the application of cyclic mechanical stretch. In validation experiments, bioprinted skeletal muscle tissues were subjected to cyclic mechanical stimulation (12% strain, 0.5 Hz, 5 h/day for 2 days), and biological assays showed that dynamic culture enhanced gene expression of early and late myogenic markers (*MyoD*, *MyoG*, *MyH1*) compared to static conditions. Given the versatility of the platform, the presented approach could be applied for the culture of cardiac bioprinted tissues.

### 6.2. Electrical Stimulation

Bioreactor platforms for electrical stimulation of hydrogel constructs rely on a commonly shared architecture, based on the application of an electric field between two parallel electrodes immersed in the culture medium. Exploiting the conductivity of the culture medium, the complexity of the setup is reduced, as no grasping or holding of the construct is needed. Setups based on modified cell culture dishes housing carbon rod electrodes have been used for stimulating cardiac constructs based on fibrin gel hydrogels [186], gelatin hydrogels [187] and GelMA/fibrin [188], obtaining improved organization of sarcomeres, establishment of gap junctions, increased calcium-handling capacity and propagation of pacing signals.

A significant platform exploiting electrical stimulation is the “Biowire”, first introduced in [166], which is based on the use of a surgical suture, around which a collagen hydrogel embedding the cells is polymerized. The structure enhances cell alignment and, by combining it with a setup for electrical stimulation, increased myofibril ultrastructural organization and elevated conduction velocity were observed [166,189].

Recently, the development of conductive hydrogels has provided an additional tool to increase the potential of electrical stimulation. Li et al. [190] developed a graphene oxide (GO)-alginate-gelatin bioink with improved printability and cell support, demonstrating enhanced viability of adipose-derived stem cells compared to conventional hydrogels. Similarly, Wang et al. [191] created an electroactive GelMA-DF-PEG hydrogel incorporating carbon nanotubes (CNTs), achieving dual crosslinking through Schiff-base reactions and UV photocuring. The CNT-enhanced hydrogel exhibited improved printability at 37 °C and conductivity (>10^−2^ S/m), suitable for electrically responsive tissues. The addition of conductive nanomaterials (GO and CNTs) improved mechanical properties, electrical conductivity, and cell interactions, promoting their use in cardiac tissue engineering applications. In this regard, when applied to hiPSC-CMs embedded in bioprinted conductive hydrogels based on dECM and multiwalled CNTs, electrical stimulation enhanced the contractile behavior of the hiPSC-CMs and promoted cell maturation [192]. Notably, Li and colleagues compared the effects of electrical stimulation on CMs cultured on GelMA hydrogels and on GelMA hydrogels incorporating dopamine-reduced graphene oxide (GelMA-PDA-rGO) [193]. The conductive GelMA-PDA-rGO hydrogels had greater cytocompatibility and promoted higher expression of gap junction proteins compared to pure GelMA hydrogels.

### 6.3. Combined Electromechanical Stimulation

In an effort to increase biomimicry of in vitro cardiac tissue models, innovative designs have been developed with the aim of providing combined electromechanical stimulation on 3D hydrogel constructs. Most setups for combined stimulation exploit the addition of electrical stimulation to setups for auxotonic mechanical loading.

In 2012, Lasher and colleagues developed fibrin hydrogel constructs seeded with rat cardiac cells on silicone posts and suspended the structures above carbon rods placed in Petri dishes [186]. After 12 days of electrical stimulation, the volume fraction of CMs was nearly double in stimulated engineered tissues compared to non-stimulated tissues. Moreover, electrical stimulation increased intercellular connections, as indicated by the connexin 43-positive membrane staining. More recently, a similar setup was used to stimulate bioprinted constructs suspended between silicone posts, resulting in increased expression and alignment of contractile proteins, as well as enhanced development of Z-lines and gap junctions [194].

The previously mentioned “Biowire” platform was upgraded in 2019, with the development of the “Biowire II”, which uses two flexible wires to suspend a fibrin hydrogel construct, providing auxotonic loading and, as its predecessor, is combinable with electrical stimulation [195]. By combining directed cell differentiation with electrical field conditioning, researchers were able to obtain atrial and ventricular tissues, and bizonal tissues with distinct atrial and ventricular ends. Subsequently, the same approach was used to obtain healthy and fibrotic tissue models and, notably, a heteropolar integrated model with fibrotic and healthy cardiac tissues coupled together [196]. Recently, the “Biowire II” platform was proposed as a model for preclinical drug evaluation. Researchers encapsulated a mixture of hiPSC-CMs and human ventricular CFs in fibrin-based hydrogel, generating arrays of microtissues. An experimental group was treated with Angiotensin II (Ang II) to establish a disease model of Ang II-induced progressive cardiomyopathy. After prolonged treatment for up to three weeks, Ang II induced a negative inotropic response and caused pronounced fibrotic remodeling [197]. At the end of the treatment period, the platform was used to assess the effects of three anti-fibrotic compound candidates, revealing distinct drug-specific effects on force profile, electrical properties, fibrotic and hypertrophic remodeling.

Zhang and colleagues [198] developed a platform integrating soft, conductive microelectrodes fabricated via high-resolution 3D printing using a conductive elastomeric ink, enabling both mechanical support and electrical stimulation of engineered cardiac tissues. The tissue model was based on a fibrin hydrogel seeded with hiPSC-CMs, which self-assembled around the flexible micropillars. Electrical pacing at a frequency of 2 Hz improved tissue alignment and synchronous contraction.

In view of high-throughput analyses, several groups have developed solutions for providing electromechanical stimulation to multi-well plates. The Eschenhagen group presented in 2014 a platform based on silicone posts and carbon plate electrodes, mountable on a 24-well plate [199]. When coupled to a video-optical system, the platform allowed monitoring the contractility of constructs over time by observing the deflection of the flexible posts. The system was used to investigate whether sustained electrical stimulation improved properties of constructs based on neonatal rat heart cells or hiPSC-CMs embedded in fibrin/Matrigel hydrogels, obtaining higher forces and higher CM density in the center of stimulated constructs, and remarkably improved sarcomere ultrastructure. In 2019, the Vunjak-Novakovic group published a setup compatible with a 12-well plate, based on silicone posts and carbon rod electrodes [200,201]. With this setup, researchers developed a protocol for driving accelerated maturation of hiPSC-CMs encapsulated in a fibrin hydrogel, while monitoring tissue properties by measuring contractile function and responsiveness to electrical stimuli. The concept was later expanded with the development of the “milliPillar” platform, which allows the parallel culture of up to six microtissues, suspended between silicone posts and electrically stimulated by parallel carbon electrodes embedded in the platform base [202]. The versatility of the platform was validated through long-term culture of CMs derived from different iPSC lines in combination with different types of stromal cells embedded in collagen or fibrin hydrogels. To streamline the analysis of force generation and calcium flux, the “milliPillar” platform’s capabilities have then been coupled with an open-source software purposely developed by the same group (“BeatProfiler” [203]). The “BeatProfiler” software processes brightfield, phase contrast or calcium imaging videos and applies machine learning algorithms for phenotyping and classification of disease and dose-dependent drug responses in the microtissues. Finally, with the purpose of recapitulating the interdependency of organ functions, the platform has been integrated into a multi-tissue chip platform, in which heart, liver, bone, and skin tissue niches are linked by recirculating vascular flow underneath [204]. In detail, each microtissue is cultured in a silicone chamber (with the “milliPillar” being the heart chamber), separated from the common vascular flow by a selectively permeable barrier. The platform was used for over 4 weeks of culture, with and without the barrier, to evaluate the pharmacokinetics of doxorubicin and investigate the miRNA responses in tissues cultured in isolation versus fluidically interlinked tissues. In this context, Kah and colleagues proposed a similar platform based on PDMS pillars, with different dimensions and composition to tune mechanical stiffness, coupled with electrical stimulation [205]. Engineered skeletal and cardiac muscle microtissues were created using C2C12 myoblasts and neonatal rat ventricular CMs, embedded in collagen I–Matrigel or collagen I alone, respectively. The tissues self-assembled between the flexible pillars into aligned, striated 3D constructs. Electrical stimulation induced active contractions, while static forces were inferred from pillar deflection. Results showed that the active force scaled with environmental stiffness following a power–law relationship, and that the tissues remained mechanoresponsive even after β-parvin knockdown, suggesting that contractile adaptation is an inherent cellular property, independent of absolute force output.

Despite the significant advancements to the field provided by the mentioned studies, some limitations and open questions remain. Most of the described setups provide either electrical or mechanical stimuli, while only a few systems allow culturing 3D models under controlled, combinable electrical and mechanical stimulations. Mechanical stimulation protocols are often oversimplified, particularly for cardiac constructs embedding flexible posts providing auxotonic loading, that lack the actual workload experienced by the myocardium in vivo. The application of cyclic loading through controlled mechanical systems increases the biomimicry of the setup, but at the cost of increased complexity. In this regard, the architecture based on posts is suitable for hydrogel constructs, but different technical solutions should be developed to actively stretch 3D bioprinted constructs with peculiar shapes and structural features. In 2023, Yadid and colleagues developed a dynamic tissue loading platform using a cantilever made of a magneto-responsive hydrogel [206]. Using permanent magnets on a linear motor system, they controlled the mechanical preload and delivered electrical stimulation through platinum electrodes. Results on engineered myocardial tissues cultured under electrical stimulation (12 V pulses at 0.5 Hz) demonstrated preload-dependent contractile responses, with baseline measurements showing force increases proportional to the cantilever pre-stretch. Moreover, treatment with digoxin (3 μM), a positive inotropic agent, produced steeper preload-dependency curves, reflecting enhanced contractility compared to untreated tissue.

Towards the realization of more relevant in vitro models, systems should accommodate variability in both rate and amplitude through modifications to control programs without any major hardware changes. First, bioreactors should incorporate live monitoring of the culture environment and construct features. Then, coupled with feedback control, they should allow automated adaptive culture, leading to optimized processes. Some steps have already been made in this direction, with the implementation of non-destructive characterization methods in bioreactors [183,199,202]. As a notable example, in 2025, Zhang et al. developed a flexible beam-based microelectrode array integrated with oriented PCL nanofiber scaffolds to culture hiPSC-CMs [207]. The flexible PDMS beams provided mechanical stimulation by deforming in response to CM contractions, simulating physiological afterload, while the embedded microelectrodes enabled non-invasive electrophysiological monitoring.

A recent study described an electromechanical stimulation bioreactor capable of real-time characterization of the force developed by a single construct and dynamic adaptation of mechanical load [208], but technical challenges still limit the development of mid- or high-throughput adaptive platforms [209]. The development of machine learning algorithms for rapid characterization of cell and construct maturation could help close this gap [203,210,211,212,213]. By training the algorithm during preliminary experiments and subsequently coupling it with stimulation control software, bioreactors could provide physical stimulation tailored to the maturation stage of the constructs, enabling native-like conditioning. Such smart bioreactor systems will allow operator-independent cultures and maximize process reproducibility and efficiency, ultimately leading to bioprocess standardization beyond the current state of the art.

## 7. Bioprinted Cardiac Tissues

**Figure 3:**
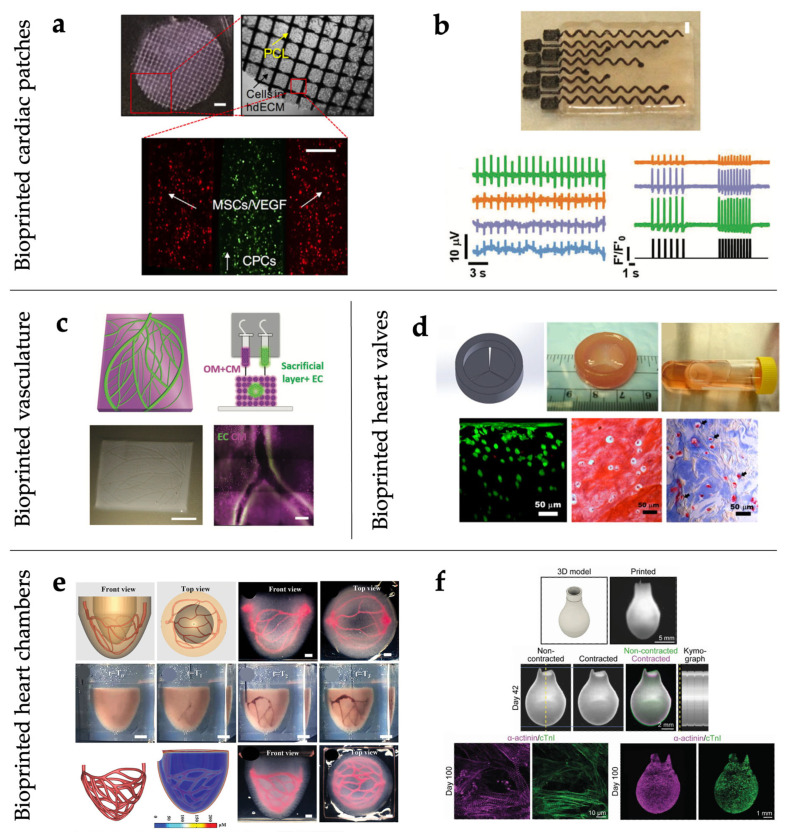
Various bioprinted cardiac constructs. (**a**) Bioprinted cardiac patch with two types of cell-laden bioink and PCL supporting layer. Adapted with permission from [224]. © 2016 Elsevier Ltd. (**b**) Bioprinted electronic cardiac patch containing ECM hydrogel and 8 electrodes. Bottom: extracellular potentials of cardiac cells from 4 distinct locations (left); calcium transients from 3 distinct locations after pacing with the printed electrodes, pacing pattern in black. (right). Adapted with permission from [229]. © 2021 The Authors. Advanced Science published by Wiley-VCH GmbH. (**c**) Bioprinted thick cardiac patch with blood vessels. Top (from left to right): 3D model; a side view of the printing concept. Bottom (from left to right): as-printed vascularized cardiac patch; CMs and ECs in the iPSCs-derived cardiac patch. Adapted with permission from [45]. © 2019 The Authors. Published by WILEY-VCH Verlag GmbH & Co. KGaA, Weinheim. (**d**) Bioprinted cardiac valve conduit with human aortic VICs. Top (from left to right): 3D model; as-printed valve conduit; intact valve conduit after 7-day static culture. Bottom (from left to right): Live/Dead image; Safranin-O staining; Masson’s Trichrome staining, with arrows indicating the newly secreted collagen around VICs. Adapted with permission from [53]. © 2013 Acta Materialia Inc. Published by Elsevier Ltd. (**e**) Perfusable ventricle construct fabricated by SPIRIT. Top (from left to right): 3D model of the hierarchical vascular network within a ventricle construct (front and top view); optical images showing the perfusion of the vascular networks (front and top view). Middle: image sequence showing the printing of the ventricle and hierarchical vascular network. Bottom (from left to right): 3D model of a dendritic vascular network with higher density; simulation of oxygen distribution within the ventricle; optical images of the printed ventricle with a densely packed vascular network, containing red dye (front and top view). Adapted with permission from [243]. © 2023 Wiley-VCH GmbH. (**f**) Bioprinted functional ventricle model. Top (from left to right): 3d model; as-printed ventricle model; Middle: contraction of a printed ventricle at day 42. Bottom (from left to right): detail view (confocal) and overview maximum intensity projections of z-stacks of printed ventricles at day 100. Adapted with permission from [49]. © 2023 The Authors. Advanced Materials published by Wiley-VCH GmbH.

Due to the steadily increasing burden of cardiovascular diseases, in recent decades many studies have focused on the development of engineered human cardiac tissues, which promise to revolutionize the treatment of failing hearts. Despite the challenges associated with the clinical use of these technologies, in the future they should provide a valid alternative to more conventional strategies such as synthetic, autologous, and allogeneic grafts, which show some main limitations in terms of material and design compatibility with the host. To date, various constructs mimicking specific components of the cardiovascular system (e.g., valves, vessels, and patches) have been fabricated using bioprinting techniques, with the aim of investigating cellular mechanisms and drug effects in vitro and repairing or replacing heart components in vivo (Figure 3) [214,215].

### 7.1. Cardiac Patches and Tissue Constructs

The ischemic event occurring during a myocardial infarction (MI) leads to the formation of a scar tissue in the affected area, since the massive death of terminally differentiated CMs leaves the heart with less contractile elements. An optimal approach to treat myocardium injuries should either promote the proliferation of resident CMs in vivo or exogenously provide new myocytes to replenish the heart. Cellular therapy, based on the direct injection of cells into the infarcted tissue, has emerged among the strategies to restore cardiac function, but it still suffers from inefficient retention, survival, engraftment and differentiation of transplanted cells [216,217].

To overcome these issues, cardiac tissue engineering has been investigated as an alternative approach to integrate cells with biomaterial structures capable of retaining them. In particular, 3D bioprinting enables the biofabrication of viable and precisely organized cardiac tissue analogs that, once implanted, could promote the migration of functional cells to the infarcted site and attract native progenitor cells for endogenous regeneration, while also providing mechanical support as traditional cardiac patches do [218,219]. Ideally, bioprinted cardiac patches should mimic the cardiac ECM for rapid integration with the host tissue, be biologically active, pre-vascularized, electrically conductive, mechanically robust, and elastic [52,215].

The inclusion of dECM within bioink composition represents one of the early successful attempts to recreate a natural microenvironment inside cardiac patches, mimicking the native tissue. Pati et al. [66] and Das et al. [67] observed enhanced structural maturation of rat myoblasts and neonatal CMs, respectively, when printed inside structures with dECM from porcine heart rather than collagen alone. Bejleri et al. [70] developed a bioprinted patch with a grid design containing cardiac dECM and GelMA for delivering pediatric human CPCs. dECM-GelMA patches revealed increased cardiogenic gene expression and angiogenic potential compared to GelMA alone. These constructs remained attached to rat hearts after epicardial placement and integrated properly with the native tissue after two weeks in vivo, as a vascular network formed to support the implanted cells. Park et al. [69] investigated the possibility of improving the therapeutic potential of human bone marrow-derived mesenchymal stem cells (BM-MSCs) via in vivo priming with engineered hepatocyte growth factor-expressing MSCs (HGF-eMSCs). They loaded a BM-MSC/HGF-eMSC mixture into a dECM patch and implanted it in a rat MI model to test this priming strategy. Patches were constructed by extruding the cell-laden dECM bioink onto disk-shaped PCL supporting frameworks and exposed to photo- and thermal crosslinking. The in vivo results indicated that primed BM-MSCs survived longer within the 3D patch and enhanced vascular regeneration via sustained secretion of paracrine factors, ultimately improving heart function and restoring the injured myocardium. In a major step toward native ECM biomimicry, Edri et al. [220] engineered patient-specific bioinks from small tissue biopsies. A piece of omentum, a highly vascularized fatty tissue, was extracted from the patient. ECM and cells were separated, with the ECM processed into a personalized thermoresponsive hydrogel and the cells reprogrammed into iPSCs. The undifferentiated cells were encapsulated within the hydrogel and, as proof of concept, differentiated into desired phenotypes, such as CMs and endothelial cells (ECs) for cardiac applications.

Since cardiac tissue contains various interacting cell types, many studies have aimed to engineer multicellular cardiac patches with greater complexity. Naturoshi Hibino’s group published a series of papers [221,222,223] detailing a novel method to create a 3D bioprinted cardiac tissue without the use of biomaterials, by assembling multicellular cardiospheres composed of hiPSC-CMs, HUVECs and human adult ventricular CFs. A bioprinter was used to pick up cardiospheres through vacuum suction and load them individually onto a needle array. Cardiospheres were allowed to fuse for 72 h before decannulation and then cultured further to fill residual gaps and form an intact patch. By varying the hiPSC-CM:HUVEC:CF ratios, they noticed that a minimum number of endothelial cells or fibroblasts was essential for cardiosphere formation, but an excess of fibroblasts blocked electrical propagation, highlighting the need for cell-ratio optimization. When implanted onto rat hearts, viable cells and erythrocytes were observed, suggesting vascularization [222]. Later in vivo experiments in a rat MI model showed improved survival rate, vessel density in the infarct area, and cardiac function in patch-treated rats [223]. Similarly, Roche et al. [48] printed vascularized cardiac spheroids, made of iPSC-CMs, human coronary artery endothelial cells (HCAECs) and human CFs, in alginate/gelatin hydrogels to form multicellular constructs. Transplanted patches showed strong functional improvements in MI mouse models and a gene expression profile resembling that of healthy mice.

Multicellular incorporation can also be achieved using multi-head printing systems. Kumar et al. [57] aimed to simulate heterocellular coupling between CMs and CFs, key to cardiac muscle contraction, since CFs can act as conductors bridging gaps between groups of CMs. Human CMs and human adult fibroblasts were loaded into two bioinks (with the same fibrinogen and furfuryl-gelatin composition), printed in a grid, and crosslinked in two steps. Connexin 43 staining confirmed CM-CF coupling via adhesion junctions.

Other works used similar alternating grid patterns to combine CMs with ECs to encourage patch vascularization. Jang et al. [224] developed a pre-vascularized stem cell patch using two bioinks, based on a dECM-vitamin B2 gel mixed with CPCs and MSCs supplemented with VEGF, respectively (Figure 3a). PCL was used to print a two-layered supporting structure. The dual cell spatial patterning of the patch induced a potent angiogenic response after subcutaneous implantation in mice: blood vessels with visible red blood cells appeared inside the patch. Maiullari et al. [58] built a custom microfluidic printing head for the simultaneous extrusion of multiple bioinks. iPSC-CMs and HUVECs were precisely compartmentalized within each strand of hydrogel (alginate and PEG-fibrinogen-based). The bioinks were co-extruded with CaCl_2_ and UV-crosslinked. After 7 days of in vitro culture and two weeks in vivo, abundant blood vessels were observed in the explanted constructs.

The hypoxic environment in the MI region due to vascular injury compromises cell survival in the early stages of patch transplantation. As an alternative or complement to patch pre-vascularization, oxygenated bioinks were explored. To meet the oxygen demand of implanted constructs, Erdem et al. [225] formulated a GelMA bioink supplemented with calcium peroxide (CPO), an effective oxygen-releasing agent [226]. CPO decomposes into hydrogen peroxide (H_2_O_2_) and calcium hydroxide, with H_2_O_2_ breaking into oxygen and water. This formulation increased the viability and metabolic activity of printed CMs and fibroblasts under hypoxic conditions. However, the use of CPO requires careful optimization, as high concentrations can lead to excessive H_2_O_2_ production during decomposition, inducing oxidative stress and cytotoxicity. To mitigate this risk, catalase was included to degrade H_2_O_2_ safely. Designing oxygen-releasing inks requires balancing oxygenation and oxidative damage, which remains a critical challenge for clinical translation.

Most cardiac bioinks are electrically insulating, leading to poor electrical propagation and electrophysiological dysfunction, which limits the regenerative capacity of the patch in vivo. Therefore, conductive components like gold nanoparticles, CNTs and GO flakes were incorporated in bioinks to improve performance. Zhu et al. [227] created a nanocomposite bioink made of alginate and gold nanorod (GNR)-incorporated GelMA and printed neonatal rat ventricular CMs and CFs in a grid pattern. These constructs showed increased levels of connexin 43 at day 14 and higher synchronized contractile frequency than pristine GelMA/alginate ones, demonstrating that GNRs can form conductive bridges within the matrix, facilitating electrical coupling between adjacent cardiac cell bundles. Additionally, the enhanced contractile behavior may also stem from the intrinsic ability of GNRs to inhibit excessive proliferation of CFs. Izadifar et al. [52] printed MeCol loaded with functionalized CNTs and HCAECs between CNT-reinforced alginate strands, forming a two-layer rhomboid mesh. The addition of CNTs imparted a highly interconnected nanofibrous morphology to both collagen and alginate, reminiscent of the Purkinje fiber network in the ventricular subendocardium. The resulting hybrid implant exhibited increased stiffness, enhanced conductivity within the physiologically relevant frequency range, and promoted HCAEC elongation and alignment. Mehrotra et al. [112] added CNTs to silk fibroin/GelMA/polyethylene glycol di-methacrylate ink encapsulating HUVECs, which was later seeded with neonatal CMs. The presence of CNTs significantly enhanced the expression of gene markers associated with mature, well-coupled CMs (e.g., connexin 43, cardiac troponin T, alpha myosin heavy chain) and increased the beating frequency of the resulting vascularized cardiac patches. Mei et al. [228] leveraged the excellent electrical and mechanical properties of GO to develop a conductive fibrin hydrogel patch. GO flakes were first allowed to adhere to MSCs before incorporation into the hydrogel. This strategy enhanced gap junction protein expression and cell adhesion through ECM protein adsorption, while also providing antioxidant protection at the infarct site, altogether improving MSC survival. Asulin et al. [229] printed a multimaterial cardiac patch with built-in stretchable electronics for electrical stimulation and extracellular signal recording (Figure 3b). Three inks were extruded: a dECM-based hydrogel containing neonatal rat cardiac cells for the tissue, a suspension of conductive graphite flakes in liquid PDMS for the electrodes, and a mixture of liquid PDMS and surfactant for dielectric insulation. The system successfully recorded extracellular potentials from multiple sites within the patch and modulated its contraction rate, an important feature for synchronizing the construct with native heart tissue post-implantation.

Finally, matching the mechanical properties of the native myocardium is essential to prevent dissection or buckling. Izadifar et al. [230] explored how bioprinting pattern influences the electrical and mechanical performance of cardiac implants. By maintaining constant strand size, hydrogel composition, and crosslinker concentration, and varying only interstrand spacing and strand alignment angle, they observed changes in both electrical conductivity and elastic modulus in HCAEC-laden alginate patches.

Overall, bioprinted cardiac patches have consistently demonstrated improved cardiac function and reduced remodeling post-MI in animal models. Outcomes include increased left ventricle ejection fraction [48,223,224], cardiac output [223], contractile area [224], and ventricular wall thickness [217,224], along with reduced end-diastolic volume [217], end-systolic volume [217], left ventricle dilation [224], and area of fibrosis [48,69,217,223,224].

### 7.2. Vasculature

Being the heart a highly vascularized organ, a biomimetic 3D bioprinting approach should consider the incorporation of functional microvascular networks inside cardiac constructs. The presence of perfusable vessels facilitates the supply of vital gases and nutrients and the removal of waste substances, particularly when dealing with thick printed tissues, since the diffusion of oxygen and nutrients is limited to small distances (100–200 μm) [231].

There are various strategies to form vasculature using bioprinting technology. One approach is printing vascular cells in strands within engineered tissues and exploiting signals from surrounding cells, growth factors and/or matrix proteins to make them self-assemble into vessel-like structures with a hollow lumen [58,224], as mentioned in the previous paragraph.

Other works focused on encapsulating sacrificial filaments inside cell-laden hydrogels to yield a network of perfusable conduits after their removal. Using this method, Noor et al. [45] created thick vascularized cardiac patches by extruding two cellular bioinks: a thermoresponsive personalized hydrogel made of omental dECM, used to print the parenchymal tissue, and gelatin, used as the sacrificial material (Figure 3c). The omentum bioink was mixed either with iPSC-CMs or neonatal rat CMs, while gelatin was mixed with either iPSC-ECs or a combination of human ECs and fibroblasts. To fit the scaffold to a patient’s left ventricle, the patch size and large vessel geometry were tailored using computer-aided design (CAD) and anatomical data coming from computerized tomography (CT) images. Smaller blood vessels were added to the design according to a mathematical model. Upon incubation at 37 °C, the blood vessel-forming cells adhered to the edges of the personalized bioink, while gelatin became liquid and was removed from the construct, leaving open, cellularized lumens ~300 mm in diameter between CM layers. Kolesky et al. [51] constructed a thick vascularized tissue within a silicone customized perfusion chip. First, a cell-laden ink containing gelatin and fibrinogen and a fugitive ink containing Pluronic F-127 and thrombin were printed onto the chip, then a fluid ECM (with the same composition as the cell-laden ink plus thrombin and transglutaminase) was cast over them. Thrombin induced fibrinogen rapid polymerization into fibrin both in the cast matrix and, through diffusion, in the printed ink, while transglutaminase slowly crosslinked gelatin and fibrin to impart the mechanical and thermal stability needed for long-term perfusion. The fugitive ink was removed by cooling, triggering its gel-to-fluid transition. This process yielded a pervasive network of interconnected channels, which were lined with HUVECs and perfused for extended periods using an external pump. The same group later developed a sophisticated biomanufacturing method named “sacrificial writing into functional tissue” (SWIFT) [46]. It is a form of embedded 3D bioprinting that relies on patterning a sacrificial gelatin ink within a living matrix characterized by a high cell density, composed of hundreds of thousands of compacted organ building blocks (OBBs). For cardiac applications, the OBB matrix was made of hiPSC-CMs and CFs spheroids, mixed with ECM and fibroblast. Initially, they fabricated cardiac tissue constructs containing a single branching channel, which, after 8 days of perfusion, exhibited pervasive sarcomeric structure and a 20-fold increase in contractility. Once the functionality of these simplified models was established, they used SWIFT to generate more complex geometries. Patient-specific anatomical data were used to bioprint a left anterior descending coronary artery network within a cardiac OBB matrix, cast into a mold replicating a wedge of the myocardium at a 1:2 scale.

In 2015 Hilton et al. established another embedded bioprinting technique named FRESH, standing for “freeform reversible embedding of suspended hydrogels” [232]. Unlike SWIFT, FRESH consists of suspending hydrogel inks in a thermoreversible support bath of gelatin microparticles, designed to maintain the intended 3D structure during the print process and be melted away afterwards. They FRESH printed to scale a part of the right coronary artery vascular tree using alginate and magnetic resonance imaging (MRI) data. It was confirmed that the tortuous bifurcations were well formed and that a wall thickness of <1 mm and lumen diameters of 1–3 mm were achieved. This approach was later improved by the same group in a publication by Lee et al. [233]. This update, termed FRESH v2.0, improved filament resolution by using gelatin microparticles with smaller diameter, uniform spherical morphology, and reduced polydispersity. They demonstrated that FRESH v2.0 could produce patent small coronary artery-scale tubes from collagen, with an inner diameter of 1.4 mm and wall thickness of ~300 μm. Most impressively, they created a CAD model of the vascular tree of a human heart using MRI data for large veins and arteries and a computational method for smaller vessels. A selected subregion was printed at adult human scale with collagen, whose self-assembly was driven by rapid pH change. Perfusion of the multiscale vasculature confirmed patency down to ~100 μm in diameter.

Another technique for creating hollow tubes is coaxial bioprinting, which directly yields tubular structures during fabrication. Gao et al. [74] produced a bio-blood-vessel using a hybrid bioink, containing dECM from porcine aortic tissue, sodium alginate, endothelial progenitor cells and microspheres loaded with a proangiogenic drug. This formulation was extruded through the shell nozzle, while Pluronic F-127 dissolved in a CaCl_2_ solution was simultaneously dispensed through the core nozzle. Upon contact, calcium ions from the core solution ionically crosslinked the alginate, resulting in the immediate formation of a tubular structure. After incubation at 37 °C for dECM thermal gelation, the sample was immersed in culture media to dissolve Pluronic and obtain a hollow vessel. The differentiated endothelial layer formed in vitro demonstrated its potential as a functional vascular graft. In another proof-of-concept study, Mao et al. [47] fabricated 3D pre-vascularized cardiac constructs using coaxial electrohydrodynamic (EHD) bioprinting. This technique integrates inkjet printing with electrospinning by applying an electric field to the printing nozzle, which draws out the bioink while minimizing shear stress on the cells. A coaxial nozzle was used to simultaneously extrude an outer bioink of alginate containing H9C2 cardiac cells and an inner bioink of collagen/calcium chloride solution with HUVECs. Instantaneous crosslinking upon contact resulted in the formation of core–sheath hydrogel filaments.

### 7.3. Heart Valves

Heart valves regulate blood flow ensure unidirectional circulation by opening and closing in response to pressure changes within the heart. Valvular heart disease, particularly affecting the aortic valve, often necessitates prosthetic replacement. Beyond traditional mechanical and bioprosthetic valves, tissue-engineered heart valves (TEHVs) have emerged as a promising therapeutic alternative. TEHVs offer potential advantages such as host integration through cell infiltration, eliminating the need for anticoagulation therapy, and the ability to grow and adapt with the patient. Bioprinting represents one strategy to fabricate functional TEHVs [234,235,236].

One of the pioneering efforts in this field came from Butcher’s group. In 2012, they started bioprinting heterogeneous valve conduits with anatomically accurate architectures reconstructed from micro-CT scans of porcine aortic valves [237]. Human aortic root smooth muscle cells (SMCs) and porcine aortic valve interstitial cells (VICs) were separately encapsulated in gelatin/alginate hydrogel to realize the valve root section and the three leaflets, respectively. The final structure was ionically crosslinked in a CaCl_2_ solution. After 7 days of culture, the encapsulated cells remained highly viable and expressed α-SMA and vimentin proteins, with VICs showing higher expression of vimentin than α-SMA, suggesting a more fibroblastic than myofibroblastic phenotype, while SMCs displayed the opposite trend. During culture, acellular hydrogels lost mechanical strength, whereas constructs containing cells maintained their tensile properties. This was likely due to ECM deposition by the cells, which compensated for the effects of alginate dissociation and gelatin release. Since the VIC phenotype is sensitive to matrix stiffness, a construct with inappropriate mechanical characteristics may promote a pathological myofibroblastic phenotype. To better control mechanical properties, the group later employed photo-crosslinking techniques [53]. In a follow-up study, they encapsulated human aortic VICs in GelMA/methacrylated hyaluronic acid (HAMA) hydrogels with tunable stiffness (Figure 3d). Increasing GelMA concentration reduced hydrogel stiffness while enhancing viscosity, which facilitated cell spreading and better supported the maintenance of the VIC fibroblastic phenotype. Based on these findings, they printed a simplified tri-leaflet heart valve model using the optimized hydrogel. Encapsulated VICs remained viable and actively remodeled the matrix by secreting collagen and GAGs. More recently, the group explored incorporating nanocrystalline cellulose into GelMA to address some limitations of GelMA for valvular applications [238], such as its inability to mimic the nonlinear stiffening critical to valvular biomechanics and its tendency to promote osteogenic differentiation of MSCs. The resulting nanocrystalline cellulose/GelMA composite exhibited nonlinear mechanical behavior and supported a quiescent VIC phenotype: human adipose-derived MSCs encapsulated in the gel showed reduced α-SMA and increased vimentin expression compared to GelMA controls. Under osteogenic media conditioning, MSCs within the composite hydrogel showed lower expression of osteogenic genes, suggesting resistance toward calcification.

Other efforts to fabricate TEHVs have leveraged the FRESH technique described earlier. Maxson et al. [239] FRESH-bioprinted a mixture of rat MSCs and highly concentrated collagen gel into a disk geometry. The aortic valve scaffold was then mounted onto a PCL supporting frame to prevent migration and folding. Although the geometry was not physiologically accurate, the primary goal of the study was to evaluate in vivo recellularization potential via subcutaneous implantation in rats. Over a 12-week period, changes in tensile properties and biomarker expression (e.g., CD163 and CD3 for immune reaction, vimentin and α-SMA for interstitial-like cell phenotypes, CD31 for endothelialization) suggested a sequence of scaffold resorption, ECM deposition, stabilization, and remodeling. Nonetheless, the mechanical strength of these bioprinted constructs remained significantly lower than that of native aortic valve cusps.

Bioprinted heart valves also hold promise for in vitro disease modeling. Aikawa’s group, focused on calcific aortic valve disease (CAVD), engineered a bioprinted model of human CAVD recapitulating individual leaflet layer-specific biomechanics [240]. First, they microdissected human valves to isolate the fibrosa, spongiosa, and ventricularis layers. Nanoindentation revealed distinct stiffness values among the layers, with fibrosa being the stiffest and spongiosa the softest. Next, they bioprinted hydrogels with varying GelMA/HAMA ratios and UV exposure times to mimic the mechanical properties of the fibrosa and spongiosa layers. VICs derived from healthy valves were encapsulated in these hydrogels and printed in a single-layered disk shape or seeded in 2D for comparison. After 14 days in osteogenic media, significant microcalcification was observed only in the fibrosa-mimicking hydrogels, which reproduced the biomechanical profile of the disease-prone layer in vivo. This demonstrated that matrix stiffness can drive pathological differentiation of VICs in vitro. Most recently, the same group leveraged intracellular proteomics and extracellular vesiculomics to further validate their fibrosa-like hydrogel model. Compared to conventional 2D cultures, the bioprinted model more accurately replicated molecular signatures of native CAVD tissue, reinforcing its potential as a disease-relevant in vitro platform [241].

### 7.4. Heart Chambers and Whole Heart Models

Over the past decade, considerable efforts have been made to generate anatomically accurate heart chambers and even whole heart models with 3D bioprinting, facing hurdles like the combination of various cell types and biomaterials in organized multiscale structures. This progress aligns with one of the ultimate goals of cardiac tissue engineering: to develop a living, pumping blood reservoir that could overcome animal models and eventually lead to therapeutic tissue grafting [78].

While a conventional EBB system requires supports to fabricate large-sized geometries with complex internal and external architectures like the heart, embedded bioprinting minimizes gravitational forces, making the process easier and more efficient [81]. Using this technique, Feinberg’s group has published three successive studies describing the FRESH printing of whole-heart constructs. These include a scaled model of a five-day-old embryonic chick heart made from alginate [232], a neonatal-scale human heart made from collagen [233], and a full-size model of an adult human heart made from alginate (approximately 8 × 10 × 12 cm^3^) [242]. Although FRESH is a cell-compatible platform, all these proof-of-concept models lack cellular components. The primary limitations to the cellularization of large tissue constructs are the extended duration of the printing process and the vast number of cells that need to be cultured. In the second-mentioned study, Lee et al. [233] also printed a cellularized left ventricle model with an ellipsoidal sandwich design: collagen was used for the inner and outer shells, while the central core region was filled with a fibrin-based gel containing human embryonic stem cell-derived CMs and CFs. These ventricles developed interconnected networks of striated CMs and showed spontaneous synchronous contractions. However, this approach, which makes use of collagen supporting walls, is not suited for enclosed heart structures with greater geometric complexity.

Beyond the complex external geometry, another key challenge in engineering large functional tissues is creating a hierarchical vascular network. Noor et al. [45] incorporated CMs and ECs in two separate bioinks to print a small-scale heart (height: 20 mm; diameter: 14 mm) with major blood vessels within a support bath. This heart model demonstrated mechanical properties similar to decellularized rat hearts, maintained compartmental integrity, and showed a homogeneous distribution of CMs one day after printing. Going beyond the conventional layer-by-layer deposition, Fang et al. [243] sought to recapitulate the heart’s complex architecture and native vasculature by developing a novel bioprinting strategy called sequential printing in a reversible ink template (SPIRIT), which bridges the gap between the previously described embedded 3D printing techniques, FRESH and SWIFT (Figure 3e). It relies on the use of a cell-laden microgel-based biphasic ink that can simultaneously act as both a bioink and a suspension medium, thanks to its shear-thinning, self-healing and photo-crosslinking capabilities. They successfully printed a vascularized ventricle model through several steps required by the SPIRIT paradigm: printing the biphasic CM-laden bioink in a suspension medium to generate the ventricle geometry, printing a sacrificial gelatin ink into the freshly printed, yet uncrosslinked, construct to generate a freeform vascular network, crosslinking the biphasic bioink, and removing the suspension medium and sacrificial ink. However, cell viability in SPIRIT remains a significant limitation due to the harsh preparation process of the bioink before printing. Furthermore, neither the studies by Noor et al. [45] nor Fang et al. [243] provided direct evidence of functional contractility or electrophysiological conduction in the generated cardiac tissues.

Kupfer et al. [244] adapted an MRI scan to obtain a simplified, scaled heart template featuring two chambers and inlet/outlet vessels extending from the top, enabling unidirectional flow. This geometry was printed inside a gelatin support bath using an optimized photo-crosslinkable bioink composed of native ECM proteins and loaded with hiPSCs. A crucial aspect in this study was the decision to induce in situ differentiation into CMs 14 days after printing. This approach allowed hiPSCs to proliferate to a sufficient density, addressing the common limitation of low cell density associated with non-proliferative, already differentiated CMs. However, the protocol for hiPSCs differentiation must be carefully optimized to reduce the risk of teratoma formation. The resulting human chambered muscle pump exhibited macroscale beating and sustained action potential propagation with responsiveness to drugs and electrical pacing, although it still lacked mature cell alignment and well-defined sarcomeric organization.

Esser et al. [49] developed a strategy to directly 3D bioprint hiPSC-CMs within a collagen-hyaluronic acid ink to fabricate centimeter-scale functional models of human cardiac ventricles (height: 14 mm; diameter: 8 mm) (Figure 3f). By day 7 post-fabrication, the constructs displayed spontaneous synchronous contractions, which persisted over long-term culture and were responsive to pharmacological stimulation with phenylephrine. By day 100, hiPSC-CMs exhibited characteristic striated patterns of *α*-actinin and cTnI, indicating structural maturation. Functional maturation was further supported by the tissues’ ability to contract against passive mechanical resistance.

## 8. Conclusions and Future Perspectives

The diverse technical solutions currently available to reproduce the structural complexity of cardiac tissue offer unprecedented flexibility for manufacturing functional cardiac components. These strategies enable the integration of both micro- and macroscale myocardial features within single bioprinting procedures and facilitate the seamless combination of cells and materials into unified, biomimetic constructs.

This review highlights several key therapeutic areas where bioprinting is expected to address critical limitations of current cardiac treatments:

(1) Cardiac valve replacement. Conventional bioprosthetic valves are primarily fabricated from animal pericardium treated with aldehydes. This material is suboptimal, particularly for patients under 60, and the only alternative remains mechanical valves, which require lifelong anticoagulation therapy. TEHVs present a promising solution but are hindered by issues such as leaflet retraction, compaction, and mechanical degradation [245].
(2) Coronary artery bypass grafting. Patients with chronic myocardial ischemia often undergo coronary artery bypass implantation using autologous vessels, typically mammary or radial arteries and the saphenous vein. While arterial grafts generally exhibit long-term patency and mechanical stability, saphenous vein grafts tend to undergo rapid post-implantation remodeling, primarily due to intimal hyperplasia, which can lead to graft failure and recurrence of ischemia. Although tissue-engineered blood vessels (TEBVs) have been under investigation for decades [246], their clinical translation remains limited due to structural and functional challenges [247].
(3) Biological patches. Patches are used in cardiac surgery to support weakened myocardium in end-stage heart failure and dilated cardiomyopathy. However, most current solutions lack cellular content, are poorly vascularized, exhibit mechanical mismatch, and do not contribute to contractility. Bioprinted cardiac patches offer the potential to overcome these limitations by integrating living cells and tailored mechanical properties [248].
(4) Biological pacemakers. Electronic pacemakers, while lifesaving, are not definitive solutions and require periodic maintenance and replacement. The identification of cells with spontaneous pacemaking activity opens the possibility of generating ‘biological pacemakers’ for lifelong rhythm management [249].

To achieve clinical and commercial viability, these designs must meet several key criteria to ensure regulatory approval and translational potential, as outlined below.

(1) Biocompatibility. All materials must comply with regulatory requirements for biocompatibility, in line with frameworks governing Advanced Therapy Medicinal Products (ATMPs) under agencies like the US FDA and EU EMA.
(2) Validation standards. Functional validation of bioengineered constructs should follow existing certification standards wherever applicable (e.g., ISO 5840-1:2021 [250] for valves, ISO 7198:2016 [251] for vascular grafts and cardiac patches).
(3) Standardized manufacturing practices. Constructs combining cells, biomaterials, and gene therapies must adhere to combined ATMP (cATMP) regulations, incorporating Good Manufacturing Practice (GMP) and Quality-by-Design (QbD) principles [252].
(4) In vivo translation. Preclinical studies in large animal models (e.g., pigs, sheep) should be conducted, when necessary, to demonstrate the therapeutic efficacy of the final construct and to obtain regulatory approval for clinical use.
(5) Immunocompatibility and safety profiling. Constructs containing animal-derived components must be rigorously tested for immunogenicity, residual decellularization reagents, and overall biocompatibility. The use of autologous patient-derived cells can reduce immune rejection risks. Comprehensive toxicology and biodistribution studies are essential to assess safety and systemic effects.

In summary, cardiac tissue bioprinting represents a transformative approach in regenerative cardiology, merging structural fidelity with biological functionality. While many challenges remain, ongoing advances in biomaterials, stem cell biology, and regulatory science are paving the way toward clinically viable solutions. Future developments will hinge not only on technological innovation but also on robust validation, manufacturing compliance, and patient-specific personalization. With sustained interdisciplinary effort, bioprinted cardiac constructs may soon become integral components of next-generation therapies for heart disease.
